# Wide-Angular Tolerance Optical Filter Design and Its Application to Green Pepper Segmentation †

**DOI:** 10.3390/s23062981

**Published:** 2023-03-09

**Authors:** Jun Yu, Shu Zhan, Toru Kurihara

**Affiliations:** 1 School of Information, Kochi University of Technology, Kami 782-8502, Kochi, Japan; 2 School of Computer Science and Information, Hefei University of Technology, Hefei 230601, China

**Keywords:** wide angular, optical filter, transfer matrix method, automatic differentiation, segmentation

## Abstract

The optical filter is critical in many applications requiring wide-angle imaging perception. However, the transmission curve of the typical optical filter will change at an oblique incident angle due to the optical path of the incident light change. In this study, we propose a wide-angular tolerance optical filter design method based on the transfer matrix method and automatic differentiation. A novel optical merit function is proposed for simultaneous optimization at normal and oblique incidents. The simulation results demonstrate that such a wide-angular tolerance design can realize a similar transmittance curve at an oblique incident angle compared to a normal incident angle. Furthermore, how much improvement in a wide-angular optical filter design for oblique incident contributes to image segmentation remains unclear. Therefore, we evaluate several transmittance curves along with the U-Net structure for green pepper segmentation. Although our proposed method is not perfectly equal to the target design, it can achieve an average 50% smaller mean absolute error (MAE) than the original design at $20^{\circ }$ oblique incident angle. In addition, the green pepper segmentation results show that wide-angular tolerance optical filter design improves the segmentation of the near-color object about 0.3% at $20^{\circ }$ oblique incident angle compared to the previous design.

## 1. Introduction

Distinguishing near-color objects—green fruits and green leaves, similar color powders, and so on—has significant applications for agriculture, farming applications, and scientific research. The radiance of the captured scene greatly impacts how objects’ surfaces are imaged and analyzed by the imaging system. Accordingly, it is feasible to design the spectral power distribution of illumination and an imaging sensor system to enhance classifying near-color objects. Previously, there was growing interest [1,2] in designing discriminative illuminates for material classification. Although their approach had compelling results under specific illumination sources, it is redistricted under controlled environments and vulnerable to intense outdoor sunlight. Yu et al. [3,4] proposed an optical-filter-based method for distinguishing near-color objects, e.g., green leaves and peppers. Their design method does not require any specific neural network; a latest neural network, such as that of [5], can be applied, and better performance is expected by using an optical filter for distinguishing near-color objects. Combing a differentiable optical filter layer with a segmentation network allows for end-to-end joint optimization from the optical filter to network weights. Such an advantage allows joint end-to-end optimization for different optics designs, e.g., phase mask design [6] and metasurface design [7].

Wide-angle imaging systems’ richer visual perception has benefited numerous domains, such as autonomous robots, self-driving, and video surveillance [8]. Although wide-angle imaging systems provide richer information, they always suffer from several problems, especially the transmitted color of light change from different angles. The optical filter is a critical component of imaging systems, which has various applications, including depth camera [9], chemical analysis [10], color imaging [11], medical imaging [12], etc. Unfortunately, the wavelength transmittance in an optical filter, especially the band-pass filter, does not always guarantee steady performance at a wide angle. Such optical filters are susceptible to wavelength shifts at a wide incident angle, leading to the color change of the object’s surface. As a result, it is not suitable for wide-angle imaging systems.

A number of studies (e.g., needle optimization [13], admittance diagram, inverse design [14,15], and reinforcement learning [16,17]) have been proposed and published that describe optical filter design for specific problems. Due to the importance of optical filters, much research in recent years has focused on designing the wide-angle optical filter to eliminate the distortion of spectral transmission of light. The band-pass optical filter is one of the most widely used optical filter types in imagining and machine vision systems. Essentially, the band-pass optical filter, in principle, is constructed by the Fabry–Pèrot interferometer. More recently, Jen et al. [18] proposed a modified Fabry–Pèrot filter design to implement a wide-angle band-pass optical filter based on a normalized admittance diagram.

**Figure 1:**
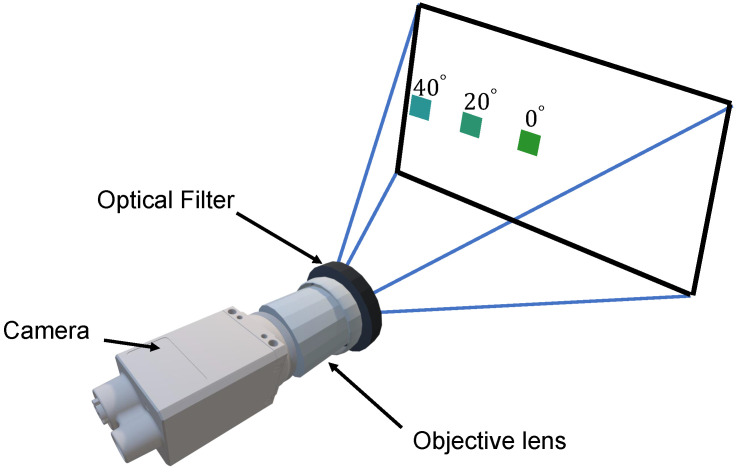
A schematic illustration of the color shift that happens in the object’s color with the angle. A green patch is used to illustrate the color shift problem caused by the optical filter. As the green patch gets closer to the edge of the field of view, its color shade changes.

Enlarging light transmission in a thin film optical filter’s pass-band while blocking out-of-band light is essential for the coated optical filter, such as dielectric filters. However, the wavelength’s transmission usually remains reliable within relatively narrow angles of view. As demonstrated by Figure 1, such non-wide optical filters are susceptible to color shift when the object is located at the edge of the field of view. In agricultural applications, the vegetation index is a vital monitoring tool for farmers and researchers. The wide-angle tolerance optical filter with a short focal length lens can provide more reliable measurement results and a larger field of view than a non-wide tolerance optical filter. Therefore, there is a great demand for wide-angular tolerance optical filter design.

Traditionally, the optical filter design is based on skill, physical intuition, and trial and error. This paper aims to develop a wide-angular tolerance optical filter design method. To achieve that, we leverage the recent advances of automatic differentiation [19] in machine learning to create the automated wide-angular tolerance optical filter design approach. In our system, each thin film layer is treated as the differential parameters in the design space. Combining the transfer matrix method [20,21] and the proposed optical merit function, we can optimize the spectral characteristics of the optical filter with respect to each thickness of the thin layers. Moreover, we also focus on the performance of the wide-angular tolerance optical filter design in computer vision problems. Specifically, we evaluate the wide-angular tolerance design transmission curve in green pepper segmentation settings.

The major technical contributions of our study are summarized as follows:

- We build the automatic wide-angular tolerance optical filter design framework. In such a framework, we model the thickness of each thin film layer as the differentiation parameters. As a result, we can utilize the autograd engine to optimize the thickness of each thin layer automatically.
- To verify the benefits of wide-angular optical filter design in applications, we connected the wide-angular transmittance curve with the segmentation network and evaluated it on the green pepper hyperspectral dataset. The wide-angular optical filter design can yield better results and demonstrate improvement in the segmentation problems.

This study is the extended version of our previous conference paper [22] by providing additional image segmentation results to verify the importance of the wide-angular optical filter design. The rest of this article is organized as follows. We provide the descriptions of the methodology in Section 2. We present numerical experiments in Section 3. Discussion is given in Section 4. In Section 5, conclusions are drawn.

## 2. Methodology

### 2.1. Transfer Matrix Method

The transfer matrix method in optics [23] is an effective and convenient mathematical method for yielding the transmission and reflectance characteristics of the thin film stack. We assume there is neither scattering nor absorption that occurs in the thin film. When an incident light enters a boundary divided by two materials that have different refractive indexes, $n_{1}$, and $n_{2}$, it leads to the transmission and reflectance of the incident light. The ratio of the incidence angle $\theta_{1}$ and the refraction angle $\theta_{2}$ can be obtained by Snell’s law:

(1) $$\begin{array}{c} \frac{\sin\theta_{1}}{\sin\theta_{2}}=\frac{n_{2}}{n_{1}}. \end{array}$$

Empirically, it can model an optical thin film as a *J* layers stack, where the specific layers can be denoted by *j*. The transmittance and reflection coefficients *t* and *r* in a boundary plane, from the *j* layer to the j+1 layer, can be estimated by the Fresnel equation. In the oblique incident, two different polarization can be expressed by applying a superscript *s* and *p* in the following equations:

(2) $$\begin{array}{cc} r_{s}^{j,j+1}=\frac{n_{j}\cos\theta_{j}-n_{j+1}\cos\theta_{j+1}}{n_{j}\cos\theta_{j}+n_{j+1}\cos\theta_{j+1}}\; & ,\;r_{p}^{j,j+1}=\frac{n_{j+1}\cos\theta_{j}-n_{j}\cos\theta_{j+1}}{n_{j+1}\cos\theta_{j}+n_{j}\cos\theta_{j+1}}\\ t_{s}^{j,j+1}=\frac{2n_{j}\cos\theta_{j}}{n_{j}\cos\theta_{j}+n_{j+1}\cos\theta_{j+1}}\; & ,\;t_{p}^{j,j+1}=\frac{2n_{j}\cos\theta_{j}}{n_{j+1}\cos\theta_{j}+n_{j}\cos\theta_{j+1}} \end{array}$$

**Figure 2:**
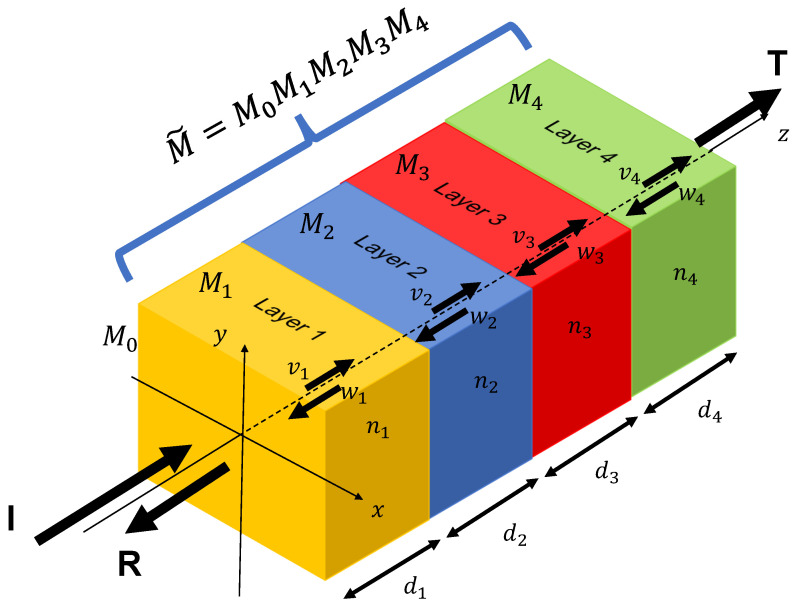
The example of an optical thin film stack comprises layer1-layer2-layer3-layer4. The *I* is the incoming incident light, and *T* and *R* represent the transmitted and reflected light. The $n_{j}$ depicts the refractive index of each layer material, and $d_{j}$ is the thickness of each layer. The transfer characteristic of each thin layer is represented by $M_{j}$.

However, as demonstrated in Figure 2, the transmission and reflection are complicated due to the multiple interfaces in the thin film stack. To simplify modeling the transmission and reflection of a thin film stack, it can use the transfer matrix $M_{j}^{\nu }$ to represent the electromagnetic wave transfer in each thin film stack at *j*-th layer for ν (*s* or *p*) polarization. Hence, the total transfer matrix of the whole thin film stack can be determined by the $\tilde{M^{\nu }}$:

(3) $$\begin{array}{c} \tilde{M^{\nu }}=\prod_{j=0}^{J-1}M_{j}^{\nu }. \end{array}$$

We assume the incident light with a relative amplitude of 1 travels from the injection layer (Air) to the thin film stack and exists in the whole thin film stack. Additionally, the coated thin layer always faces the light source, and no incident light enters the thin film stack from the substrate. The forward direction is from the incident light source to the substrate of the thin film stack. The transmitted electric field $v_{j}$ propagates from layer *j* to the next layer j+1, i.e., in the forward direction. Conversely, the reflected electric field $w_{j}$ travels in the reverse direction, i.e., in the backward direction. The relationship of $v_{j}$ and $w_{j}$ in the *j*-th layer and $v_{j+1}$ and $w_{j+1}$ in the j+1-th layer for both *s* and *p* polarization can be formulated as follows:

(4) $$\begin{array}{cc} \left(\begin{array}{c} v_{j}\\ w_{j} \end{array}\right)\; & =M_{j}^{\nu }\left(\begin{array}{c} v_{j+1}\\ w_{j+1} \end{array}\right) \end{array}$$

for j=0,1,...,J−1, where the

(5) $$\begin{array}{cc} \; & M_{j}^{\nu }=\left(\begin{array}{cc} e^{-i\delta_{j}} & 0\\ 0 & e^{i\delta_{j}} \end{array}\right)\left(\begin{array}{cc} 1 & r_{\nu }^{j,j+1}\\ r_{\nu }^{j,j+1} & 1 \end{array}\right)\frac{1}{t_{\nu }^{j,j+1}} \end{array}$$

where $\delta_{j}=d_{j}\;k_{j}$ estimates the phase of the light wave when transmitting through layer *j* with thickness $d_{j}$ and the wave vector $k_{j}$. By assigning ν as *s* and *p*, we can easily substitute Equation (2) for Equation (5) to calculate the transfer matrix $M_{j}^{\nu }$ for the *j*-th layer.

Finally, we can obtain the coefficient *r* and *t* of the whole thin film stack by

(6) $$\begin{array}{c} r_{\nu }=\frac{{\tilde{M^{\nu }}}_{10}}{{\tilde{M^{\nu }}}_{00}}\;t_{\nu }=\frac{1}{{\tilde{M^{\nu }}}_{00}} \end{array}$$

where we can substitute the *s* and *p* for ν to calculate the coefficient at an oblique incident and ${\tilde{M^{\nu }}}_{k,l}$ is the k+1-th row, l+1-th column entries of the matrix $\tilde{M^{\nu }}$. Based on the above coefficients, we obtain the reflectivity and transitivity *R* and *T* by

(7) $$\begin{array}{c} R_{s}={\left|r_{s}\right|}^{2}\;R_{p}={\left|r_{p}\right|}^{2} \end{array}$$

(8) $$\begin{array}{c} T_{s}=\frac{n_{j}\cos\theta_{j}}{n_{0}\cos\theta_{0}}{\left|t_{s}\right|}^{2}\;T_{p}=\frac{n_{j}\cos\theta_{j}}{n_{0}\cos\theta_{0}}{\left|t_{p}\right|}^{2} \end{array}$$

where the $n_{0}$ and $\theta_{0}$ denote the refractive index and angle for the incident light, and the $n_{j}$ and $\theta_{j}$ denote the refractive index and angle at the final transmitted *j*-th layer.

### 2.2. Optical Merit Function

The optical merit function is a characteristic function for measuring the discrepancy between optimization output and the target design and deriving its derivatives concerning the design space of the optical system [24]. A large variety of elements can be involved in the optical filter design. One might think of optimizing its various parameters simultaneously, e.g., materials, thickness, and structure. For simplicity and consistency with our already manufactured optical filter, we regard the thickness $d_{j}$ of each layer as our design space. It is ultimately a matter of optimizing the thickness of each thin film layer. Additionally, it can be defined as the interference effects of the incident light passing various layer boundaries in the thin film stack. As it relates to the wide-angular tolerance design, the mathematical optimization progress will be halted when it is impossible to minimize the error of the optical merit function in the particular design space.

**Figure 3:**
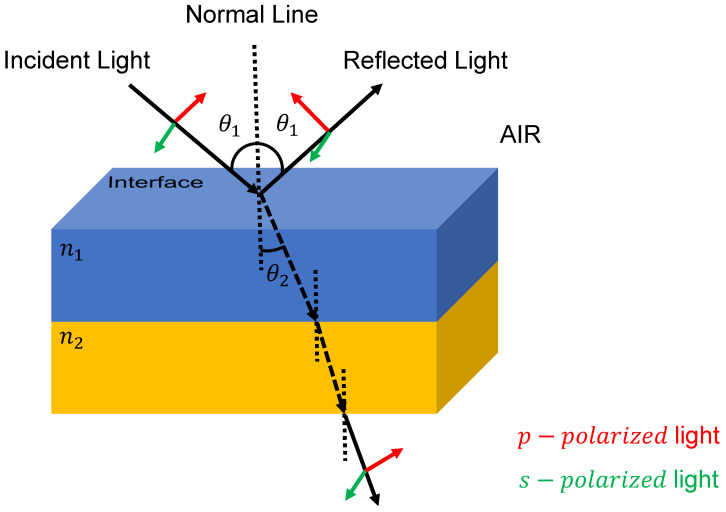
Diagram of the *p*-polarized light and *s*-polarized light. The electronic field of *p*-polarized light is parallel to the plane of the incident light, while the *s*-polarized light is perpendicular to the incident plane. For convenience, we refer the $n_{1}$ and $n_{2}$ as the refractive index for two different materials.

To devise an optical merit function for wide-angular tolerance design, we need to consider the optimization of the normal and oblique incidents simultaneously. As observed in Figure 3, when the oblique incident travels through the plane interface, it should be separated into the s-polarization and p-polarization components. Therefore, the optical merit function for wide-angular tolerance design can be written as the following equations:

(9) $$\begin{array}{c} Merit_{1}\;=\;\frac{1}{N}\sum_{\lambda =1}^{N}\left[{\left(T_{\lambda }^{\theta_{0}}\;-\;T_{\lambda }^{target}\right)}^{2}\;+\;{\left(T_{\lambda }^{\theta_{1}}\;-\;T_{\lambda }^{target}\right)}^{2}\right] \end{array}$$

where the *N* denotes the number of the wavelength over λ, the $T_{\lambda }^{\theta_{0}}$ and $T_{\lambda }^{\theta_{1}}$ can be expressed by substituting a particular incident angle, e.g., $\theta_{0}$ or $\theta_{1}$ in the following equation

(10) $$\begin{array}{c} T_{\lambda }^{\theta }=\left[T_{s}\left(d,n,\theta \right)+T_{p}\left(d,n,\theta \right)\right]/2 \end{array}$$

The $T_{\lambda }^{\theta }$ denotes the transmittance of the optical thin film stack for wavelength λ with the incident angle θ, where the $\theta_{0}$ represents the normal incident light and $\theta_{1}$ represents the oblique incident light. d∈R indicates the vector of the thickness of each layer, the n∈R indicates the refractive indices of the material in each thin layer. More broadly, we proposed the different merit function based on the oblique incident angle as follows:

(11) $$\begin{array}{c} Merit_{2}=\frac{1}{N}\sum_{\lambda =1}^{N}\left[{\left(T_{\lambda }^{\theta_{0}}-T_{\lambda }^{target}\right)}^{2}+{\left(T_{p,\lambda }^{\theta_{1}}-T_{\lambda }^{target}\right)}^{2}+{\left(T_{s,\lambda }^{\theta_{1}}-T_{\lambda }^{target}\right)}^{2}\right] \end{array}$$

With the mentioned differences, it calculates the *p* and *s* polarization independently to the target design at oblique incident light. Consequently, by using the developed optical merit function, we can optimize the optical filter design for normal and oblique incidents at the same time. Since the result of the automatic differential typically has a generated negative value, the thickness of each thin layer would be physically implausible. Inevitably, our experiment involves a non-negative constraint for each layer thickness $d_{j}$.

## 3. Results

To confirm the effect of the improvement of our proposed method, we evaluate the segmentation performance of each transmittance curve of different merit functions, as mentioned in the previous section. First, we report the optimized transmittance curve at an oblique incident angle by different merit functions. Next, we evaluate the performance of the green pepper segmentation for each transmittance curve at both normal and oblique incident angles. The evaluation is carried out by a segmentation neural network on the synthesized RGB images, replacing each transmittance curve.

### 3.1. Optimized Transmittance Curve

**Figure 4:**
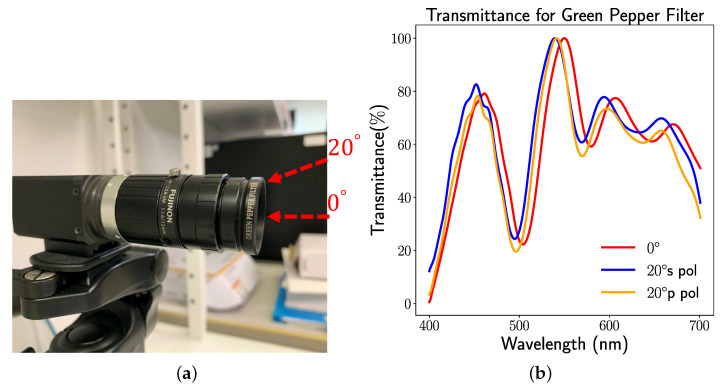
(**a**) The fabricated Green Pepper Filter mounted on a machine vision camera system. (**b**) The transmittance curve of the Green Pepper Filter at different incidence angles.

As illustrated in Figure 4, the Chroma Technology Corporation successfully fabricated the optical filter designed by our previous proposal method Optical Filter Net [3] for green pepper segmentation. Figure 4a demonstrates our fabricated optical filter with a machine vision camera system. We called our fabricated optical filter a Green Pepper Filter. Our Green Pepper Filter was made for the M25.5×0.5 filter thread. The camera in our system is the LUCID Vision Triton 5.0 Camera equipped with a 2/3 inch Sony IMX264 CMOS sensor [25]. The objective lens is FUJINON HF12 XA-5M 1:1.6/12mm. As shown in Figure 4b, our Green Pepper Filter cannot guarantee the stability of the transmittance curve for the oblique incident. Our implemented optical thin film comprises the $Ta_{2}O_{5}$ (refractive index $n_{1}=2.3$) and $SiO_{2}$ (refractive index $n_{2}=1.46$), and the layer number is 60, whereas the thickness of each thin layer of the Green Pepper Filter is unknown to us. To facilitate implementing our wide-angle optical filter design, we follow similar parameters setting with our already implemented optical thin film, e.g., the same materials and same number of layers. Optical glass (refractive index $n_{1}=1.52$) is chosen as a substrate in our experiment. The whole structure of our optical filter is based on an elemental type thin film structure, which can be described by {Air|2.3|1.46|…|2.3|1.46|Optical Glass}.

Our multi-layer thin film aims to generate a target transmission spectral for both normal incident and oblique incident angles. The design space is the thickness of each layer. In particular, our proposed wide-angular tolerance design is achieved by optimizing the thickness of thin layers by the TMM-fast library [21]. The TMM-fast library is built on the PyTorch library and Autograd differential engine. In the optimization process, we set the thickness of each layer as the differential tensor. Therefore, the TMM-fast library turns the traditional transfer matrix method into a differentiable optics simulator for the optical filter, which allows us to find the optimal results in the design space. We used the constrained L-BFGS-B [26] optimization algorithm in the SciPy Library [27] in our experiment.

**Figure 5:**
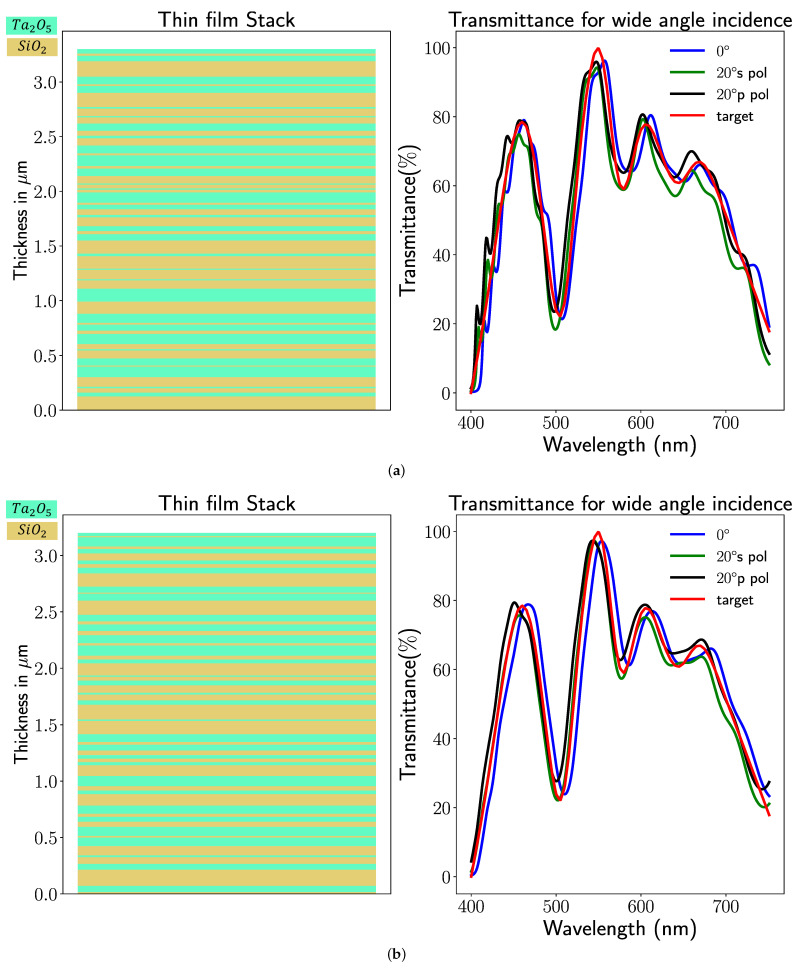
The thickness optimization result and its corresponding simulated transmittance for (**a**) merit function 1 and (**b**) merit function 2.

To quantify the difference between our method and target design, we utilize the mean absolute error (MAE) as a metric in our experiment. In Table 1, we show the comparative results of the manufactured optical filter and the optimized results by two optical merit functions. Apparently, both proposed merit functions achieve similar transmittance curves to our target design at the oblique incident. Meanwhile, by observing the transmittance curve derived from each merit function (see Figure 5), merit function 2 can obtain a smoother curve than merit function 1.

### 3.2. Green Pepper Segmentation

**Figure 6:**
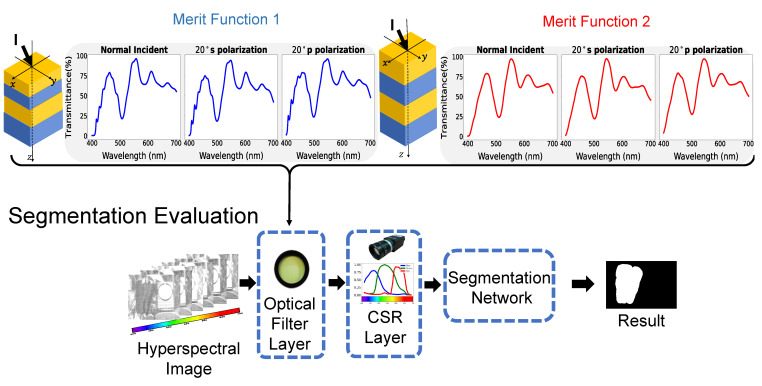
The illustration of the evaluation method for our Green Pepper Filter, merit function 1 optimization result, merit function 2 optimization result at $0^{\circ }$ and $20^{\circ }$. In the whole pipeline, the weights of the neural network and camera layer is fixed. We replace different transmittance curve of the different design for the weight of optical filter layer and evaluate the segmentation performance on the green pepper hyperspectral dataset.

Our experimental setup is schematically demonstrated in Figure 6. We adopt a baseline deep neural network structure, e.g., a U-Net structure [28], for evaluating the effectiveness of an optical filter in green pepper segmentation since it is commonly used for image segmentation. The whole end-to-end deep neural network structure is trained with the transmittance curve from our prior proposal [3]. Then we freeze segmentation network weights and replace the weight of the optical filter layer with the transmittance curve of the Green Pepper Filter and each transmittance curve of the wide-angular tolerance design for both normal and oblique incident angles. Therefore, all evaluation results are derived with the same segmentation network. Furthermore, to verify the effectiveness of the optical filter, we conduct a comprehensive experiment with a no-filter setting. In this setting, the weights of the optical filter layer are kept at 1 for the whole wavelength. Both weights in the optical filter layer, as well as the camera spectral response (CSR) filter layer, are fixed. Only the segmentation network is trained on the same dataset, i.e., achieving the best segmentation for a standard RGB camera without a filter.

**Figure 7:**
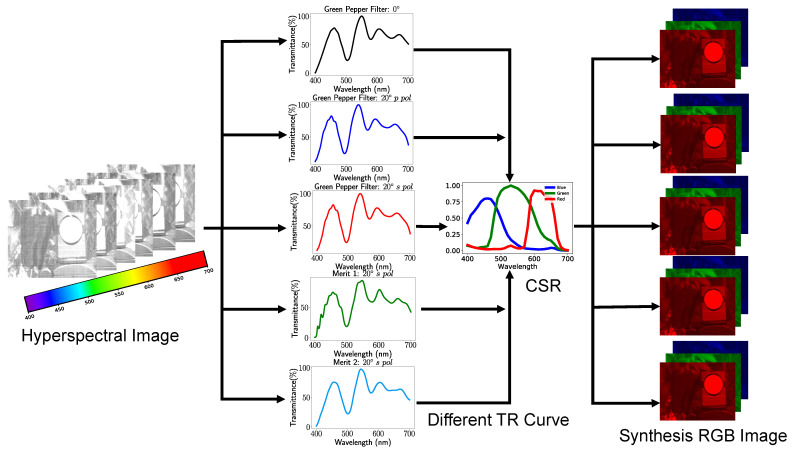
The above figure demonstrates the details of how to replace different transmittance curves in the optical filter layer. As a result, a different RGB feature map was generated by putting the hyperspectral image into the optical filter layer and CSR layer.

One of the critical insights in our previous study [3] is to model the transmittance curve of the optical filter as one parametric neural network layer, connecting those layers with the segmentation neural network (i.e., U-Net structure for green pepper segmentation) and ultimately utilize back-propagation to refine the transmittance curve of the optical filter in a hyperspectral image dataset. The whole optical system, optical filter with the RGB camera, can be accurately simulated by optical filter layer with the CSR layer. The synthesized RGB image can be considered a three-channel feature map inside the deep neural network structure. To compare each transmittance curve, we replace each of them in the optical filter layer respectively and evaluate the segmentation performance. As demonstrated in Figure 7, we fix the weight of the camera spectral response layer and segmentation network for fair comparisons. We change the weight of the optical filter layer with different transmittance (TR) curves of the already implemented optical filter and proposal merit functions 1 and 2 at $0^{\circ }$ and $20^{\circ }$. We adopt the camera spectral response (CSR) from the Lucid Triton 5.0 machine vision camera [25].

The hyperspectral image dataset is from our previous study, which was captured in the Green House by NH2-TKT hypersepctral camera (EBA JAPAN Co., Ltd., Tokyo, Japan). Quantitative evaluation results of different optical filter design are shown in Table 2. The metric for evaluating the green pepper segmentation is mean intersection over union (mIoU) [29], which is commonly used for semantic segmentation evaluation. It is specified as the intersection between the predicted segmentation map and the ground truth, divided by the union between the predicted segmentation map and the ground truth:

(12) $$\mathrm{mean}\;\mathrm{IoU}=\frac{1}{k}\sum_{i=1}^{k}\frac{P_{i}\cap G_{i}}{P_{i}\cup G_{i}},$$

where *k* denotes the number of the classes, and $P_{i}$ and $G_{i}$ represent the predicted segmentation maps and the ground truth for each class *i*, respectively.

**Figure 8:**
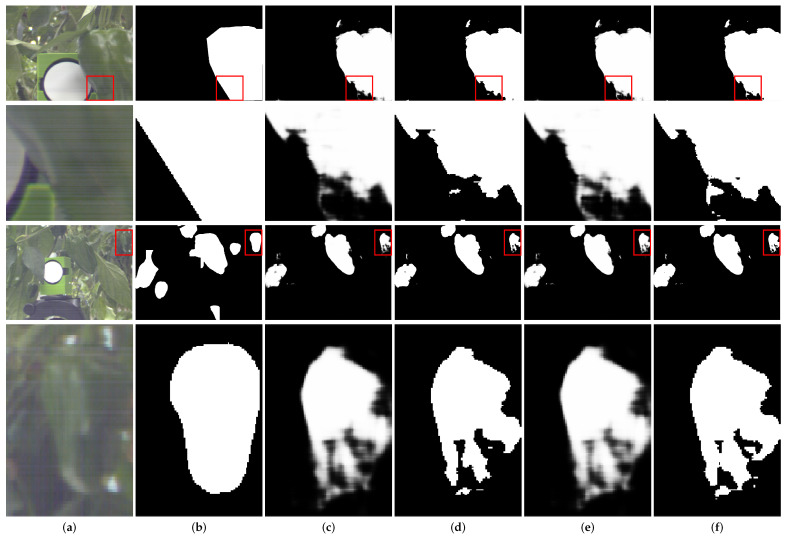
Visual comparison on segmentation results. The red box in rows 1 and 3 are enlarged in rows 2 and 4, respectively. (**a**) The RGB image and relate patch details. (**b**) Ground truth. (**c**) Prediction of simulated Green Pepper Filter. (**d**) Binary prediction of simulated Green Pepper Filter. (**e**) Prediction of merit function 2. (**f**) Binary prediction of merit function 2. Note that our proposed merit function can improve the green pepper segmentation results.

As there is no color change in the no-filter setting, the normal and oblique incidents retain the same performance in the no-filter setting. As shown in Table 2, our proposed wide-angular tolerance optical filter design can achieve a better result than the Green Pepper Filter at an incident angle of $20^{\circ }$, while keeping the same performance at the normal incident angle. For further comparison, we applied $20^{\circ }$*s*-polarization TR curve of Green Pepper Filter and Merit Function 2 to the optical filter layer in our end-to-end network structure and evaluated the segmentation results in Figure 8. Compared to the Green Pepper Filter segmentation results, our proposed method can achieve better segmentation results of the input images. It may be reasonable to suppose that our proposed method can improve the deep neural network segmentation performance at oblique incident angle. We note that merit function 2 can achieve more close TR curves to the target design at the oblique incident. Therefore, the end-to-end segmentation network with merit function 2 can achieve better results than merit function 1 for both *p* and *s* polarization at the $20^{\circ }$ oblique incident angle. In addition, it is apparent that the Green Pepper Filter and our proposed merit function obtain better results than no filter settings.

## 4. Discussion

We present an exploration of using a differentiable framework to solve the wide-angular tolerance optical filter design and verify the improvement at broad angle incident in our optical filter design for specific computer vision tasks, e.g., image segmentation. We examine how performance improvement related to wide-angular tolerance optical filter design can advantageously affect deep neural networks focused on similar color object segmentation, e.g., green pepper segmentation.

Our results provide clear evidence that wide-angular tolerance optical filter design can be driven from the transfer matrix method with the specific merit function by the Autograd differential engine. However, our design space is still limited in thickness for each layer. As stated in the previous section, multiple factors are related to the optical filter design and fabrication. It would be beneficial to utilize multiple factors to enhance the optical filter design and coatings. Yeung et al. [30] proposed a global deep learning-based inverse design framework based on a generative adversarial network [31] to utilize multiple factors for the reverse design. We are interested in future studies to see how to combine those factors together for further improvement. Applying the novel physical-based deep learning solver [32] is another direction.

Despite the benefits of the transfer matrix method, drawbacks remain. It is restrained to the linear, homogeneous thin film stack and limited geometrical structures. Therefore, a further study focusing on incorporating a deep neural network-based inverse design [33] with some basic optical filter structures is suggested. It is known in the literature [34] that some fundamental thin film stack orders exist, e.g., band-pass filter, long-pass filter, and Fabry–Perot filter design. Integrating those basic filter structures as prior identification might be possible to facilitate optical filter design. Lastly, from the optical coating point of view, the thin film design should be robust to the fluctuations in spectral response caused by a slight thickness variation in each thin layer.

## 5. Conclusions

In this work, we introduced a novel automatic differentiation-based model to design wide-angular tolerance optical filter. Using the differentiable model and optical merit function we built, we can search for an optimal optical filter design in the limited design space for normal and oblique incidents. To the best of our knowledge, our study is the first to integrate differentiable models and optical merit functions for designing wide-angular tolerance optical filters. Moreover, with the recent development of computational resources, our proposed automatic differentiation-based framework can be adapted to refine the optimal design in much large design space.

Throughout our experiments, this work also examined the impact of wavelength shift in the optical filter at an oblique incident angle on deep neural networks for similar color object segmentation. We illustrated that the deep neural network structure with our proposed wide-angular optical filter design improves the green pepper segmentation results in our hyperspectral datasets. Additionally, we are also interested in extending our wide-angular tolerance design for more applications in wide-angle imaging systems. We believe our proposed wide-angular tolerance optical filter design method will be useful for other tasks targeted by combining wide-angular imaging systems and deep neural networks. In the future, we will continue working on improvements in the wide-angular tolerance design of the optical filter. Other future work aspects include the deep-learning-based inverse design for optical filter design and so on.

## Figures and Tables

**Table 1:** Mean absolute error of the Green Pepper Filter and the proposed methods compared to the target design.

	Method	Green Pepper Filter	*Merit*_1_	*Merit*_2_
Angle				
0°		0.0035	0.0445	0.0592
20° *s* polarization		0.0875	0.0498	0.0292
20° *p* polarization		0.0994	0.0484	0.0403

**Table 2:** The evaluation of segmentation result of different design.

Method	Incident Angle		mIoU
Green Pepper Filter	$0^{\circ }$		0.882
	$20^{\circ }$	*s* pol	0.879
		*p* pol	0.875
Merit 1	$0^{\circ }$		0.883
	$20^{\circ }$	*s* pol	0.881
		*p* pol	0.878
Merit 2	$0^{\circ }$		0.883
	$20^{\circ }$	*s* pol	0.882
		*p* pol	0.879
No filter	$0^{\circ }$/$20^{\circ }$		0.829

## Data Availability

Data sharing not applicable.

## Abbreviations

The following abbreviations are used in this manuscript:

Ta${}_{2}$O${}_{5}$	Tantalum Pentoxide
SiO${}_{2}$	Silicon Dioxide
TR	Transmittance curve
MAE	Mean Absolute Error
mIoU	Mean Intersection over Union
CSR	Camera Spectral Response
